# Is waist circumference a better predictor of blood pressure, insulin resistance and blood lipids than body mass index in young Chilean adults?

**DOI:** 10.1186/1471-2458-12-638

**Published:** 2012-08-10

**Authors:** Macarena Lara, Patricia Bustos, Hugo Amigo, Claudio Silva, Roberto J Rona

**Affiliations:** 1 PhD Programme of Public Health, Faculty of Medicine, University of Chile, Independencia, 1027, Santiago, Chile; 2 Department of Nutrition, Faculty of Medicine, University of Chile, Independencia, 1027, Santiago, Chile; 3 School of Public Health, Faculty of Medicine, University of Chile, Santiago, Chile; 4 Department of Psychological Medicine, King’s College, London, UK

**Keywords:** Waist circumference, body mass index, cardiovascular risk factors, young adults

## Abstract

**Background:**

It has been reported that waist circumference (WC) is a better predictor of cardiovascular risk factors than body mass index (BMI), although the findings have not been consistent. The aim of this study was to assess which measurement, BMI or WC, is more strongly associated with blood pressure, homeostatic model assessment (HOMA) and blood lipids in young Chilean adults.

**Methods:**

999 subjects aged 22 to 28 years were randomly selected from a registry of individuals born between 1974 and 1978 at the Hospital of Limache, Chile. Weight, height, WC, blood pressure, HOMA and lipoproteins were assessed in a cross-sectional study.

**Results:**

In multivariable regressions BMI and WC were associated with blood pressure, HOMA and lipoproteins at similar level of explained variation (R^2^ between 1.6 % for Low Density Lipoproteins (LDL) and 15.6 %, the highest for HOMA and triglycerides) and similarly OR in standardised logistic regressions between 1.1 (95 % CI: 0.9 and 1.4) for LDL and 2.9 (95 % CI: 2.4 and 3.4) for elevated HOMA. When both WC and BMI were included in the model collinearity was high and only for HOMA was there a small independent contribution of each index (R^2^ = 1 %); for other outcomes the pattern was inconsistent.

**Conclusion:**

The strength of the associations of WC and BMI for any cardiovascular risk factors was similar, but highest for HOMA and triglycerides. WC and BMI are equally useful for monitoring the consequences of obesity in young adults.

## Background

As obesity is a risk factor for type 2 diabetes, hypertension, cardiovascular disease, dyslipidaemias and some types of cancer [1] it is important to choose the best measure of obesity to monitor its effects in populations.

Body mass index (BMI) has been the most used index to assess obesity [1]. However, lately it has been suggested that waist circumference (WC), waist hip girth ratio, and WC height ratio are better measures of obesity than BMI in predicting cardiovascular risk factors [2-7]. It has been said that visceral fat, which is more correlated with WC than subcutaneous fat, has a direct link with the liver and greater lipolytic activity increasing the level of free fatty acids and decreasing insulin activity. In addition, visceral fat increases low density lipoproteins (LDL) and decreases high density lipoproteins (HDL) [8]. In spite of the plausibility of this explanation, reports remain controversial because studies have not been consistent in showing the advantage of WC over BMI [9-12].

The great majority of the studies assessing which measure of obesity is best have included a wide age range and age has been treated as a confounder [3-5,9-12]. There is little evidence that the published results would be applicable to young adults. This is a serious drawback as obesity increases markedly during the second and third decade of life [13,14] and health practitioners need appropriate tools to advise young adults about the consequences of obesity. In relation to specific risk factors, few studies have assessed the measures of obesity in relation to insulin resistance [15]. The great majority have used a diagnosis of type 2 diabetes [5,9-11]. Such an approach limits the value of the evidence because type 2 diabetes is usually uncommon until late adulthood and would render any assessment less sensitive in youngsters; most individuals with high insulin resistance are not yet diabetic. Finally, few of the studies present their evidence in relation to the three main risk factors, namely, blood pressure, insulin resistance and blood lipids in the same population. It is important to study these points in order to learn about the consistency of the value of each obesity measure in relation to each risk factor and to compare their relative effect sizes in the same population.

In a study of young adults in Chile homeostatic model assessment (HOMA), blood pressure and blood lipids were assessed. The aim of this study is to assess whether BMI or WC is a better measure of obesity in relation to important cardiovascular risk factors in young adults.

## Methods

### Study population and design

Limache is a semi rural town of approximately 40 000 inhabitants (49 % men), located 108 km from the capital of Chile. Agriculture is the main economic activity and the poverty rate is similar to that of the country.

This is a cross-sectional study of 999 adults (437 men and 562 women) aged between 22 and 28 years. The initial sample for the study of risks of cardiovascular disease was 1050 individuals, 250 fewer than for our asthma study [16]. These subjects were selected by simple random sampling from a sampling frame of 3096 newborns registered between 1974 and 1978 in the hospital of Limache, Chile [16]. 260 of the 1050 subjects (approximately 24.8 %) were randomly replaced because they were unavailable for examination, including 75 (seven per cent of the total sample) who were unwilling to participate. A further 51 (4.9 %) out of the 1050 selected subjects, after inclusion of the replacement fraction, did not consent to participate thus the final sample for analysis was 999. The addresses of participants were collated using information from the National Health Service, the National Registry and, if the participants had changed address, from relatives at the old address or neighbours. The participants were visited at home whenever possible. No data were missing for anthropometric measurements, blood pressure measurements and laboratory assessments.

This study was approved by the Ethics Committee of the Faculty of Medicine of the University of Chile. All individuals signed an informed consent form to participate in this study.

### Collection of information

#### a) General and anthropometric characteristics

Socio-demographic information was collected using a questionnaire and was usually administered in the participant’s homes, whereas anthopometric measurements, blood pressure and a blood specimen were obtained by trained university nurses in the Limache hospital or a local health facility.

Weight was measured using electronic calibrated SECA scales to the nearest 100 grams. The subjects were weighed barefoot, wearing minimal clothing (T-shirt and trousers or blouse and skirt, and underwear), with the feet together in the centre of the weighing scales and the head looking forward. Height was measured using an anthropometer to the nearest 1 mm. Subjects stood barefoot on a flat surface, with their back against the instrument, and their head in the Frankfort position. BMI was expressed in kg/m^2^. WC was measured, using an inextensible tape, midway between the lower rib and the iliac crest after exhaling with the person standing.

### b) Blood pressure, HOMA index and blood lipids

Blood pressure was taken with a digital automatic sphygmomanometer, Omron 740, with a self-inflating cuff. The mean of the last two of three blood pressure measurements was used for this analysis.

Blood samples, obtained following 12 hour fasting, were processed and frozen at the Limache hospital for analysis in the Laboratory of Nutrition of the Pontificia Universidad Catolica of Chile. Total cholesterol was measured using the enzymatic colorimetric method (Gesellschaft für biochemica und diagnosed Germany mbh), high density lipoprotein (HDL) by the precipitation technique of Seigler and Wu, and plasma triglycerides by the enzymatic method with HUMAN clarification factor (Gesellschaft für biochemica und diagnosed mbh Germany). Low-density lipoprotein (LDL) was calculated using the Friedewald formula [17].

The HOMA index was calculated by the formula: (insulin x glycaemia)/22.5 (insulin expressed in *u*U/ml and glucose in mmol/L) [18]. Glycaemia was assessed by the enzymatic colorimetric method *(GOD/PAD method, human diagnosed, Germany)*, and plasma insulin by radioimmunoassay (Insulin kit, DCP, Los Angeles, USA). As there is no international threshold definition of insulin resistance, a HOMA ≥2.53, equivalent to 1 standard deviation above the average HOMA value in 19 to 40 year olds in Santiago, Chile was used [19].

### Analysis

Statistical analyses were performed using the STATA software version 10.1.

First, using multiple linear regression analyses, we assessed the contribution of BMI and/or WC to each dependent variable (systolic blood pressure, diastolic blood pressure, HOMA index, total cholesterol, HDL, LDL and triglycerides) unadjusted and adjusted for age, sex, tobacco consumption and number of belongings (number of domestic appliances in working order in the household: refrigerator, gas-fuelled water heating, personal computer, washing machine and microwave oven). The effects of BMI and WC were assessed using the partial regression coefficients *β* (95 % Confidence Interval (CI)) and the percentage of explained variation attributable to each main factor (R_BMI_^2^ and R_WC_^2^). The residuals of each model were assessed for normality and homocedasticity.

Second, we performed multiple logistic regressions based on dependent binary variables: systolic blood pressure ≥140 mm Hg, diastolic blood pressure ≥90 mm Hg [20], HOMA index ≥2.53 [19], total cholesterol ≥200 mg/dl, HDL ≤40 mg/dl, LDL ≥160 mg/dl and triglycerides ≥150 mg/dl [21]. Odds ratios (OR) were calculated separately for the contribution to BMI and WC in relation to each binary dependent variable. As BMI and WC use different units they were standardised in Z-scores, based on the mean and one standard deviation of BMI and WC respectively.

We also assessed two way interactions between sex and each independent variable on the response variable to decide if stratification by sex was necessary. We presented results separated by sex only when interaction was significant at p <0.01 or if the p-value was between 0.05 and 0.01, and the plotting of relevant values were considered relevant.

## Results

### a) Characteristics of the study population

The age of the sample ranged between 22 and 28 years, men were slightly older on average, but women had a slightly higher mean number of years of full education (Table 1). Mean BMI was high, over 25, in both sexes but higher in women and WC was higher in men. Mean HOMA was similar to the threshold for insulin resistance. Blood pressure and plasma triglycerides were higher in men and total cholesterol, HDL and LDL levels were higher in women. Smoking was common with a prevalence of over 50 %; high blood pressure was uncommon, but significantly higher in men. A third of the population was insulin resistant and about half of the sample showed low levels of HDL cholesterol, more commonly in men (Table 1).

**Table 1:** Prevalence and distribution of cardiovascular risk factors and socio-demographic factors by sex

**Variable**	**Total**		**Men**		**Women**		p *
	**(n=999)**		**(n=437)**		**(n=562)**		
	**Mean**	**SD**	**Mean**	**SD**	**Mean**	**SD**	
Age (years)	24.8	1.6	24.9	1.6	24.7	1.6	0.022
Full time education (years)	11.1	2.8	10.9	2.8	11.3	2.7	0.033
Number of belongings	1.5	0.95	1.44	0.96	1.54	0.95	0.118
BMI (kg/m^2^)	25.8	4.5	25.3	3.7	26.2	5.0	0.002
WC (cm)	83.9	11.3	85.1	9.6	83.0	12.5	0.003
Systolic blood pressure (mm Hg)	114.6	13.5	123.5	11.4	107.8	10.7	< 0.001
Diastolic blood pressure (mm Hg)	72.5	8.8	75.7	8.4	70.0	8.3	< 0.001
HOMA	2.53	1.58	2.59	1.84	2.48	1.34	0.276
Total cholesterol (mg/dl)	178.3	38.4	175.6	36.0	180.5	40.0	0.047
HDL (mg/dl)	41.4	11.7	39.5	11.1	42.9	11.9	< 0.001
LDL (mg/dl)	114.6	35.6	111.1	33.4	117.3	37.1	0.007
Triglycerides (mg/dl)	112.5	69.4	125.3	81.7	102.5	56.2	< 0.001
**Variable**	**%**		**%**		**%**		***p*†**
Smoking	57.8		66.8		50.7		< 0.001
Systolic blood pressure ≥140 mm Hg	4.3		8.9		0.7		< 0.001
Diastolic blood pressure≥90 mm Hg	3.4		5.3		2.0		0.004
HOMA ≥2.53	34.5		35.7		33.6		0.495
Total cholesterol ≥200 mg/dl	25.9		24.9		26.7		0.812
HDL ≤40 mg/dl	49		55.1		44.3		0.003
LDL ≥160 mg/dl	10.4		7.8		12.5		0.056
Triglycerides ≥150 mg/dl	17.6		22.4		13.9		< 0.001

SD is standard deviation and % percentage.

* p value estimated in an analysis of variance (ANOVA).

† p value estimated using chi square test.

### b) Results from multiple linear regression analyses

Significant interactions were found in the multiple linear regressions between sex and both measures of obesity (BMI and WC) on triglycerides (p = 0.002 for both). Thus results are given separately by sex for triglycerides.

BMI and WC were significantly associated with the blood pressure levels, HOMA and blood lipids when analyzed separately, after adjusting for age and sex. R_BMI_^2^ and R_WC_^2^, proportion of variation explained, were very similar for each outcome but the R_BMI_^2^ was slightly higher for blood pressure and R_WC_^2^ was higher for HDL. R^2^ was high for HOMA (15.6 %) in comparison to any other dependent variables (Table 2). Adjustment for tobacco use, number of belongings and sex did not change the level of association shown in Table 2 (data not shown).

**Table 2:** Association between BMI and/or WC and blood pressure, HOMA and blood lipids adjusted by age and sex

	***β*coefficient (95 % CI)**^a^					
	**Systolic blood pressure (mm Hg)**	**Diastolic blood pressure (mm Hg)**	**HOMA**	**Total cholesterol (mg/dl)**	**HDL (mg/dl)**	**LDL (mg/dl)**
**BMI only in the model**						
BMI (kg/m^2^)	0.7	0.7	0.14	1.4	−0.7	1.0
	(0.6 to 0.9)	(0.5 to 0.8)	(0.12 to 0.16)	(0.9 to 1.9)	(−0.8 to −0.5)	(0.5 to 1.5)
R_BMI_^2^^b^	0.058	0.109	0.156	0.025	0.068	0.016
**WC only in the model**						
WC (cm)	0.3	0.2	0.06	0.5	−0.3	0.4
	(0.2 to 0.3)	(0.2 to 0.3)	(0.05 to 0.06)	(0.3 to 0.8)	(−0.4 to −0.2)	(0.2 to 0.6)
R_WC_^2^^c^	0.049	0.098	0.156	0.025	0.081	0.016
**BMI and WC included in the model**						
BMI (kg/m^2^)	0.7	0.5	0.07	0.7	−0.002	0.5
	(0.3 to 1.0)	(0.3 to 0.8)	(0.02 to 0.12)	(−0.6 to 2.0)	(−0.4 to 0.4)	(−0.7 to 1.7)
R_BMI_^2^^b^	0.009	0.012	0.007	0.001	0	0
WC (cm)	0.03	0,1	0.03	0.3	−0.3	0.2
	(−0.1 to 0.2)	(−0.05 to 0.2)	(0.01 to 0.05)	(−0.2 to 0.8)	(−0.4 to −0.1)	(−0.3 to 0.7)
R_WC_^2^^c^	0	0.001	0.007	0.001	0.013	0.001

^a^ The statistical analysis excluded triglycerides in this table because there was a significant interaction of measures of obesity and sex on triglycerides.

^b^ R_BMI_^2^ proportion of variation explained by BMI.

^c^ R_WC_^2^ proportion of variation explained by WC.

When BMI and WC were both entered into the model for each dependent variable, adjusted for age and sex, the only outcome that both variables contributed independently to was HOMA. BMI, but not WC, was associated with systolic and diastolic blood pressure, while WC, but not BMI, was associated with HDL (Table 2). However, R_BMI_^2^ and R_WC_^2^ values were minimal in this analysis indicating a high level of collinearity between these BMI and WC.

BMI and WC were associated at a similar level with plasma triglycerides in men and women after adjustment for age (Table 3). Only WC made a small independent contribution to triglyceride level when BMI and WC were included together. Adjustment for tobacco use and number of belongings did not change the levels of association shown in Table 3 (data not shown).

**Table 3:** Association between BMI and WC and plasma triglycerides by sex adjusted by age

	***β*coefficient (95 % CI)**	
	**Triglycerides (mg/dl)**	
	**Women**	**Men**
**BMI only in the model**		
BMI (kg/m^2^)	4.2	7.3
	(3.4 to 5.1)	(5.3 to 9.3)
R_BMI_^2^^a^	0.140	0.107
**WC only in the model**		
WC (cm)	1.8	3.0
	(1.4 to 2.1)	(2.2 to 3.7)
R_WC_^2^^b^	0.150	0.120
**BMI and WC included in the model**		
BMI (kg/m^2^)	1.4	1.7
	(−0.8 to 3.5)	(−2.9 to 6.3)
R_BMI_^2^^a^	0.002	0.001
WC (cm)	1.3	2.4
	(0.4 to 2.1)	(0.6 to 4.1)
R_WC_^2^^b^	0.012	0.013

^a^ R_BMI_^2^ proportion of variation explained by BMI.

^b^ R_WC_^2^ proportion of variation explained by WC.

Residuals in all the multiple linear analysis were normally distributed.

### c) Results from logistic regression analyses

**Figure 1:**
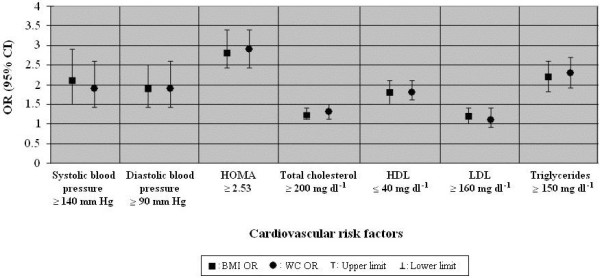
**Odds Ratio (OR) of cardiovascular risk factors associated with 1 standard desviation increment of BMI and WC adjusted by age and sex.** There were no differences between the effects of BMI and WC for any of the outcomes in the analysis.

As we did not find two way significant interactions between sex and BMI or WC on each dependent variable (p values ≥0.01) analyses were carried out for the total sample. Standardised BMI and WC were associated to a similar degree with each of the dependent variables. The associations were stronger for HOMA and lowest for total cholesterol and LDL (Figure 1). Adjustment for tobacco use, number of belongings and sex did not change the level of associations shown in Figure 1 (data not shown).

## Discussion

The strength of the association between BMI or WC and blood pressure, lipids and HOMA was similar. The two measures were associated more strongly with HOMA and triglycerides than with any other cardiovascular risk factor both in the multiple linear and logistic regression analyses. The findings from multiple linear and logistic regression analyses were consistent for each dependent variable. The independent contribution of BMI and WC when significant was small highlighting the high collinearity between these two measures.

### Interpretation of findings

The associations reported between measures of obesity and cardiovascular risk factors in this study underscore the potential for modifying the risk profile of individuals in a population. The 95 % CIs were narrow indicating that we can rely in the accuracy of our estimates. The effects were intermediate for HOMA, triglycerides and blood pressure, lower for HDL, and modest for total cholesterol and LDL. The ranking of effects for measures of obesity and dyslipidaemias were similar to those reported by Barzi and colleagues [12], though the approach used to assess the impact of measures of obesity was different to our study. We found that the association between WC or BMI and triglycerides was higher in men than women. A possible explanation would be that men have higher levels of blood triglycerides at this age which are susceptible to increase when BMI or WC increases, a finding also reported in a European study of 38 year olds [22].

A robust test to infer that BMI and WC are equally useful in the assessment of obesity was that BMI and WC made only a small independent contribution (approximately one percent) or no contribution at all to the studied outcomes when both were included in the model; BMI and WC made an independent contribution to HOMA, BMI made a contribution to blood pressure levels and WC to HDL. Others studies have also shown that the use of BMI and WC together offer only a marginal advantage [23,24], with the exception of Zhu and colleagues [25].

Our second test to show that the two measures, BMI and WC, were of similar value was that they were associated with the same strength in terms of OR and R^2^ with each of the seven outcomes. Our results are consistent with Huxley and colleagues in relation to hypertension [10] and appear consistent with a receiving operating characteristic (ROC) analysis and assessment of area under the curve [4,11], but not with NHANES findings which showed that WC was a more suitable measure than BMI [5]. Our findings regarding measures of obesity and HOMA were consistent with a metaanalysis of follow-up studies which used type 2 diabetes as outcome [9] and another two meta-analyses [10,11], despite slightly stronger associations with WC in some subgroup analyses which was also reported in a ROC analysis [4]. As in our study, the strength of association between BMI or WC and dyslipidaemia variables was found to be similar in another study [12].

Our results and many other reports show no difference between BMI and WC in the assessment of coronary risk factors [4,9,11,12]. However, there are studies that have shown an advantage of WC over BMI both slight [10,11] and meaningful [2,5,26,27]. In addition WC was shown to be more related to cardiovascular mortality than BMI in a recently published meta-analysis [28]. The variation of results between studies is large. This may be due to the study designs, the age at which BMI and WC of the participants were measured and the outcome used in these studies (risk of cardiovascular disease, a meaningful cardiovascular event or cardiovascular mortality). The main contribution of our study is that it focuses on young adults, an area that it has been rarely addressed in the literature.

Our findings suggest that in young adults BMI is as appropriate a measure of obesity as WC. We were unable to find other studies in 20–29 year olds. In older ages obesity increases, mainly in women, and the cardiovascular risk factors are more common than in young age therefore the level of association between BMI or WC and each response variable could be different [9,11,29,30]. However, our results showed levels of association similar to those reported in other studies carried out in adults with a broader age range [4,10-12]. It is also possible that some differences between studies may be due to ethnicity [10-12]. Our study is based in a group with an admixture of Spanish and native Indians and we could not make comparisons between groups.

Another important fact is that the biggest OR for each indicator was with resistance to insulin, reinforcing the idea that both excess BMI and/or WC are associated with the development of type 2 diabetes [31,32] and would support the view that insulin resistance provides an enabling environment for the subsequent development of other cardiovascular risk factors [33]*.*

### Strengths and weaknesses of this study

The strengths of this population study are: the high response rate; the reliability of the measurements taken by trained professionals; the appropriate sample size for the analysis undertaken; the assessment of the most important cardiovascular risk factors in one study; the use of insulin resistance which allowed us to include participants at high risk of type 2 diabetes in a population of young adults; and the inclusion of both multiple linear regression to assess the association throughout the range of values and logistics regression using standardization to compare the effects of BMI and WC.

The cross-sectional design of this study can be considered a weakness. However, reverse causality i.e. blood pressure level, dyslipidaemia or insulin resistance causing obesity, is implausible. Our study based on a young population could not assess major cardiovascular events and mortality.

Another limitation of our study is that we did not assess waist hip girth ratio because we did not measure hip girth. We did not include waist girth height ratio because reference values are not readily available and the most readily available are for BMI and WC.

Adolphe Quetelet proposed the BMI index approximately 175 years ago *(Sur l'homme et le développement de ses facultés, ou Essai de physique sociale. 2 volumes 1835)*. Its value as a tool to assess obesity continues despite the existence of other measures such as WC. The success of BMI is based on its simplicity and the reliability of its component measurements, the ease of use of reference values of obesity and, as demonstrated in our study, the level of association with major cardiovascular risk factors at a similar level to their association with WC.

## Conclusion

This study demonstrates that WC does not offer any advantage over BMI regarding a range of major cardiovascular risk factors; both are associated with cardiovascular risk factors with similar strength. There is no great advantage in using both measures of obesity for population monitoring as their independent contribution is only marginal. This study shows that BMI, which can be reliably measured, is as appropriate for monitoring obesity as WC. BMI could be more useful in populations where the measurement of WC might be problematic either because the available facilities do not allow privacy or because the measurement of WC is less acceptable than measurement of weight and height. It is also advantageous that the threshold values of BMI for overweight and obesity are similar for both sexes and are widely known by practitioners.

## Competing interest

The authors declare that they have no competing interests.

## Authors’ contributions

Macarena Lara and Roberto Rona proposed the original aim of the paper, the analytical approach and drafted the paper. Macarena Lara carried out the analysis. Hugo Amigo and Patricia Bustos were in charge of the planning and data collection of the Limache project and reviewed critically the versions of the paper. Claudio Silva was the statistical advisor and critically reviewed the statistical contents of the paper. All authors commented each version of the paper and approved the final version.

## Pre-publication history

The pre-publication history for this paper can be accessed here:

http://www.biomedcentral.com/1471-2458/12/638/prepub
